# The Expression of the Short Isoform of Thymic Stromal Lymphopoietin in the Colon Is Regulated by the Nuclear Receptor Peroxisome Proliferator Activated Receptor-Gamma and Is Impaired during Ulcerative Colitis

**DOI:** 10.3389/fimmu.2017.01052

**Published:** 2017-09-04

**Authors:** Anthony Martin Mena, Audrey Langlois, Silvia Speca, Lucil Schneider, Pierre Desreumaux, Laurent Dubuquoy, Benjamin Bertin

**Affiliations:** ^1^Univ. Lille, U995 – LIRIC – Lille Inflammation Research International Center, Lille, France; ^2^INSERM, U995, Lille, France; ^3^CHU Lille, Service des Maladies de l’Appareil Digestif et de la Nutrition, Hôpital Claude Huriez, Lille, France; ^4^CHU Lille, Service de Chirurgie Digestive et Transplantations, Hôpital Claude Huriez, Lille, France

**Keywords:** PPARgamma, thymic stromal lymphopoietin, intestinal epithelial cells, ulcerative colitis, colon

## Abstract

The etiology of inflammatory bowel diseases remains largely unknown. We previously demonstrated that the expression of the peroxisome proliferator activated receptor-gamma (PPARγ) is downregulated in colonic epithelial cells of patients with ulcerative colitis (UC). PPARγ is a nuclear receptor that modulates inflammation. We hypothesized that its deficiency may play a role in the loss of intestinal homeostasis through the control of immunomodulatory factors. We found that thymic stromal lymphopoietin (TSLP), an epithelial cytokine with pleiotropic functions, is regulated by PPARγ. While this cytokine possesses two isoforms, only the short form (sfTSLP) was regulated by PPARγ. sfTSLP mRNA expression was decreased both in PPARγ knock-down Caco2 cells and cells treated with PPARγ antagonist, whereas PPARγ agonists induced the expression of sfTSLP in Caco2 and T-84 cells. The response element activated by PPARγ was identified in the promoter of the sfTSLP gene by chromatin immunoprecipitation and gene reporter assays. The expression of sfTSLP was significantly decreased in the colonic mucosa of UC patients compared to controls and was correlated with PPARγ expression. Our results identified sfTSLP as a new PPARγ-target gene and support the hypothesis that, in UC, PPARγ deficiency in colonic mucosa could play a role in the loss of intestinal tolerance through an impaired sfTSLP expression.

## Introduction

In humans, the intestinal mucosal surface is an essential exchange area between the host and its environment. In the context of intestinal homeostasis, mucosal immune cells must be able to tolerate commensal and food antigens while keeping their ability to fight pathogens (1). A fundamental actor in this dialog between luminal antigens and mucosal leukocytes is the intestinal epithelial cell (IEC) (2). Although initially considered as a simple physical barrier, it is now clear that IECs actively participate to the induction and maintenance of intestinal homeostasis. For instance, IECs respond to microorganism antigens or metabolites by increasing the synthesis of mucus, the secretion of anti-microbial peptides and/or by promoting immunoglobulin A transcytosis (2). In addition, IECs have the ability to secrete cytokines and chemokines at their basal pole, thereby regulating the functions of the underlying intestinal immune cells (2). Notably, it has been demonstrated that, through the secretion of immunoregulatory molecules such as transforming growth factor-β (TGF-β), thymic stromal lymphopoietin (TSLP), or retinoic acid, IECs have the capacity to regulate the ability of dendritic cells to induce regulatory T cells (3–5).

Recent studies suggest that IEC dysfunctions may actively participate in the pathogenesis of ulcerative colitis (UC) (6, 7). UC (along with Crohn’s disease) is one of the major types of inflammatory bowel diseases (IBD) which are chronic, complex, relapsing immune-mediated diseases of the gastrointestinal tract of unknown etiology (7, 8). Our previous work has demonstrated that the expression of peroxisome proliferator activated receptor-gamma (PPARγ) is reduced in the colonic epithelial cells (CEC) of individuals with UC (9, 10). PPARγ is a nuclear receptor which plays a key role in lipid and glucose metabolisms as well as in gut homeostasis (11, 12). Conditional knock-out mice in which PPARγ expression is selectively ablated in CECs demonstrate an increased susceptibility to DSS-induced colitis (13). Conversely, PPARγ synthetic agonists inhibit inflammation and reduce disease severity both in experimental models of colitis and in UC patients (12, 14–16). These results clearly underline the importance of CEC in controlling the magnitude of the inflammatory response in the gut, at least in part, in a PPARγ-dependent manner. Despite these observations, as well as the abundance of PPARγ in the colon (17), how this nuclear receptor exerts its anti-inflammatory functions within the intestine is poorly known and most of its target genes, notably in CEC, remain to be characterized.

Our hypothesis is that PPARγ deficiency in CEC during UC might play a role in the loss of intestinal homeostasis trough the regulation of immunoregulatory factors. One possibility would be the potential role of PPARγ in the control of cytokine production by CEC. In addition, CEC express some cytokine receptors and, consequently, are able to respond to the cytokine environment within the intestinal mucosa (18, 19). The aim of this study was therefore to identify PPARγ target genes involved in CEC immune functions relevant to UC physiopathology. Our results identified the gene encoding the short isoform of TSLP (sfTSLP) as a new gene regulated by PPARγ in CEC and support the hypothesis that, in UC, the impaired expression of PPARγ in colonic mucosa could play a role in the loss of intestinal tolerance through a decrease of sfTSLP expression.

## Materials and Methods

### Cell Culture and Stimulation

Caco-2 cells (ATCC^®^ CRL-2102™) were grown in Dulbecco’s Modified Eagle’s Medium (DMEM, Invitrogen, Life Technologies, Cergy-Pontoise, France) supplemented with 20% fetal calf serum (FCS, Dutscher, Brumath, France), 1% penicillin-streptomycin (Invitrogen, Life technologies), and 1% non-essential amino acids (Invitrogen, Life technologies). T84 cells (ATCC^®^ CCL-248™) were grown in a 1:1 mixture of Ham’s F12 medium and DMEM supplemented with 5% FCS and 1% penicillin-streptomycin. H292 cells (ATCC^®^ CRL-1848™) were grown in RPMI-1640 supplemented with 10% FCS and 1% penicillin-streptomycin. Generation and maintenance of PPARγ knock-down Caco-2 cells were described in Ref. (10). All cell lines were cultured as confluent monolayers at 37°C in a controlled, 5% CO_2_ atmosphere.

For cell stimulations, 1 × 10^6^ cells per well were seeded in 6-well plates. Serum deprivation was used 16 h prior to stimulation in order to synchronize the cells. Cells were treated with GED (30 mM; Nogra Pharma Ldt, Italy), pioglitazone (10 µM; Sigma-Aldrich), 5-ASA (30 mM; Sigma-Aldrich), GW9662 (10 µM, Sigma-Aldrich), or poly I:C (10 µg/L; Invivogen). When necessary, the DMSO vehicle (Sigma-Aldrich) was used as control. After 24 h of stimulation, cells were washed twice with sterile PBS and were lysed with 1 ml of TRIzol^®^ reagent (Invitrogen™, Cergy Pontoise, France). The plates were frozen at −80°C for subsequent RNA and protein extraction. Cell stimulations were performed in 3 or 6 replicates.

Culture of polarized Caco-2 cells was done as follow: 3 × 10^4^ cells were seeded on Transwell^®^ filters in 12-well plates. During the first 7 days, complete culture medium (DMEM containing FCS) was added both to the upper and lower compartment. Cell polarization is obtained between days 8 and 21 of culture by culturing cells with DMEM containing FCS in the lower compartment and DMEM without FCS in the upper compartment. Measurement of the Trans-Epithelial Resistance (TER) was done every 2 days (before medium replacement) to check cell differentiation and integrity of the cellular barriers. At day 21, polarized Caco-2 cells were stimulated with PPARγ agonist. After 24 h of stimulation, cells were washed twice with sterile PBS and were lysed with 1 ml of TRIzol^®^ reagent.

### RNA Extraction

RNAs were isolated with the TRIzol^®^ reagent according to the manufacturer’s instructions and resuspended in RNAse-free, DEPC-water. The purity and concentration of the RNAs were evaluated by UV spectroscopy on a Nanodrop system (Nyxor Biotech, Paris, France).

### Quantitative PCR (qPCR)

Expression of genes was quantified by qPCR of corresponding reverse transcribed mRNA. 1 µg of total RNA was reverse-transcribed into cDNA using the High Capacity cDNA Archive kit (Applied Biosystems). Amplification was performed using an ABI PRISM StepOnePlus detection system (Applied Biosystem) using Power SYBR^®^ Green PCR master Mix (Applied Biosystem). Primer pairs were chosen with qPrimer depot software.^1^ See Table S1 in Supplementary Material for the oligonucleotides used in this study. Quantification of qPCR signals was performed using ΔCt relative quantification method using GAPDH as a reference gene. Values were represented in terms of relative quantity of mRNA level variation or fold increase compared to control conditions.

### Protein Analysis

Peroxisome proliferator activated receptor-gamma protein was detected from total proteins obtained after TRIzol extractions. Proteins were separated by polyacrylamide gel electrophoresis and electroblotted on nitrocellulose membrane. PPARγ was detected using a monoclonal rabbit antibody (C26H12) as primary antibody (Cell Signaling Technology^®^) and goat anti-rabbit IgG peroxydase conjugate (Jackson ImmunoResearch). Nitrocellulose membranes were probed with a mouse monoclonal anti-β actin antibody (Sigma-Aldrich^®^, France) to assess equal protein loading.

Short form TSLP protein was detected by immunoprecipitation followed by western-blot analysis. 6 million of Caco-2 cells were used for each immunoprecipitation. When indicated, cells were stimulated during 24 h in 100 mm cell culture dishes. Cells were washed and scraped in PBS buffer, then lysed in IPH buffer (50 mM Tris pH 8, 150 mM NaCl, 5 mM EDTA, 0.5% NP40, and protease inhibitors) for 1 h in ice. After centrifugation 15 min at 15,000 *g*, the supernatants were recovered and dosed. The same amount of protein was engaged in each immunoprecipitation (1.5 mg). 2 µg of TSLP antibody (Clone # 184638 rabbit polyclonal, Abcam) were added and the tubes were incubated overnight at 4°C under rotation. The next day, a mixture of protein A/G agarose beads (Santa Cruz) was added and incubation was continued 2 h at 4°C under rotation. Then, beads were washed 5 times with IPH buffer followed by denaturation in Laemli sample buffer at 95°C for 5 min. Immunoprecipitated material was analyzed by western-blot using the TSLP antibody (Clone # 184638 rabbit polyclonal, Abcam) as primary antibody and goat anti-rabbit IgG peroxydase conjugate (Jackson ImmunoResearch). β-actin protein was detected to assess equal protein engagement (1% input).

### *In Silico* Analysis of sfTSLP Promoter

Presence of PPAR response element sequences were investigated *in silico* in the promoter of the sfTSLP. 1000 base pairs upstream of the transcription initiation site were analyzed by two different softwares: NUBIScan^2^ (20) and PPRE Search^3^ (21).

### Chromatin Immunoprecipitation Experiments

The physical binding of PPARγ onto the sfTSLP gene promoter was studied by ChIP experiments in Caco-2 cells (10 × 10^6^ cells) stimulated for 24 h with 30 mM GED in 150 mm cell culture petri dishes. Caco-2 cells were synchronized by the addition of serum-free medium for 16 h and then stimulated for 24 h using the protocol described previously. Cells were then rinsed with PBS and the protein–DNA complex was fixed by adding 10% PFA for 30 min at room temperature. This binding was stopped by the addition of glycine (0.125 M). Cells were collected by scrapping in cold PBS and protease inhibitors (Sigma). The cell pellet obtained by centrifugation was taken up in 300 µl SDS buffer (1% SDS, 10 mM EDTA, 50 mM Tris–HCl pH 8, protease inhibitors + PMSF 0.1 mM) and sonicated (Diagenode, BioruptorUCD200TM-EX) for 30 s, followed by 30 s’ resting time. For each immunoprecipitation, 125 µL of crosslinked sonicated sample was diluted with 225 µL of IP buffer (1% triton X-100, 150 mM NaCl, 2 mM EDTA, 20 mM Tris–HCl pH 8.1, and protease inhibitors) and precleared for 4 h by adding 40 µL of protein A/G beads (50% slurry protein A/G Sepharose, Clinisciences) and 5 µg of salmon sperm DNA (Invitrogen). Complexes were immunoprecipitated with specific anti-PPARγ antibodies (C26H12 rabbit monoclonal antibody, Cell Signaling) by incubation overnight at 4°C under rotation. Immune complexes were recovered by adding 40 µL of protein A/G Sepharose (50%) plus 2 µg salmon sperm DNA and incubated for 4 h at 4°C. The beads were washed twice in wah buffer 1 (0.1% SDS, 1% Triton X-100, 150 mM NaCl, 0.1% Deoxycholate, 1 mM EGTA, 2 mM EDTA, 20 mM Tris–HCl pH 8.0), twice in wash buffer 2 (0.1% SDS, 1% Triton X-100, 500 mM NaCl, 0.1% Deoxycholate, 1 mM EGTA, 2 mM EDTA, 20 mM Tris–HCl pH 8.0), once in wash buffer 3 (0.25 mM LiCl, 0.5% Deoxycholate, 0.5% NP40, 0.5 mM EGTA, 1 mM EDTA, 10 mM Tris HCl pH 8.0), and 3 times in wash buffer 4 (1 mM EDTA, 10 mM Tris–HCl pH 8.0). The co-immunoprecipitated DNA was then extracted with 150 µL of extraction buffer (0.1 M NaHCO3, 1% SDS). Cross-linking was reverse overnight at 65°C. DNA was then purified using the PCR Clean-up kit (Macherey-Nagel) and analyzed by PCR.

### Cloning

Genomic DNA fragments were cloned upstream of the luciferase reporter gene (plasmid pGL4.10 [Luc2]; Promega). The fragment corresponding to the 1,000 bp upstream of the transcription initiation site of the human *sftslp* gene (construction “Prom TSLP2”) was obtained by PCR from genomic DNA of Caco-2 cells (sense oligonucleotide 5′-CGCTCGAGGGATGTCTATCCTTTGCTAAAG-3′; antisense oligonucleotide 5′-CGAAGCTTGGCGGAGGGCACTCGTCGCGAAAAG-3′). The fragment corresponding to the 548 base pairs upstream of the transcription initiation site of the human *sftslp* gene (construct “Prom TSLP2Δ1”) was obtained by PCR from genomic DNA of Caco-2 cells (sense oligonucleotide 5′-CGCTCGAGGGTACCTGAGTGGAAACTGTT-3′; antisense oligonucleotide 5′-CGAAGCTTGGCGGAGGGCACTCGTCGCGAAAAG-3′). The PCR products were cloned into a TOPO pCR4 vector (TOPO TA cloning, Invitrogen) and then sequenced in order to check for potential *Taq* Polymerase errors. For each construct, a mutation-free fragment was subcloned into the vector pGL4.10 [Luc2] (Promega) using the *XhoI*/*HindIII* restriction sites introduced into the oligonucleotides (underlined in the sequence).

### Transient Transfections and Reporter Gene Assays

Reporter plasmids and the empty vector control were transiently transfected in Caco-2 cells using Nucleofector™ Technology. For each condition, 2 × 10^6^ cells (50–70% confluency) were transfected with 2 µg of plasmids using SE 4D-Nucleofector solution and program DS-123. Transfected cells were seeded in 48-well plates at 100,000 cells per well and treated by PPARγ agonists for 24 h. Luciferase activity was measured using the luciferase assay kit (Promega) in fluorometer (FLUOstar Omega, BMG Labtech) according to the manufacturers’ instructions. Total protein contents were dosed using Bradford reagent (Euromedex) and used to normalize luciferase activity.

### Patients and Tissue

A local ethics committee (Comité de Protection des Personnes Nord Ouest IV, CHRU Lille, France) approved the study and all subjects gave informed consent (No. DC-2008-642). Surgical colonic samples were obtained from patients with an established diagnosis of UC according to international criteria and from control patients (patients underwent surgery for colorectal cancer and patients for diverticulitis of the sigmoid). The detailed characteristics of patient cohort are given in Table S2 in Supplementary Material. Colonic epithelial cells were isolated from resected colonic specimens as previously described (10).

### Statistics

The data are presented as mean with SEM or SD. All graphs were plotted and analyzed with GraphPad Prism 5 Software (San Diego, CA, USA) using a two-tailed non-parametric Mann–Whitney test. *P* values < 0.05 were considered statistically significant.

## Results

### Reduced Expression of the Gene Encoding TSLP in PPARγ Knock-Down Cells

**Figure 1:**
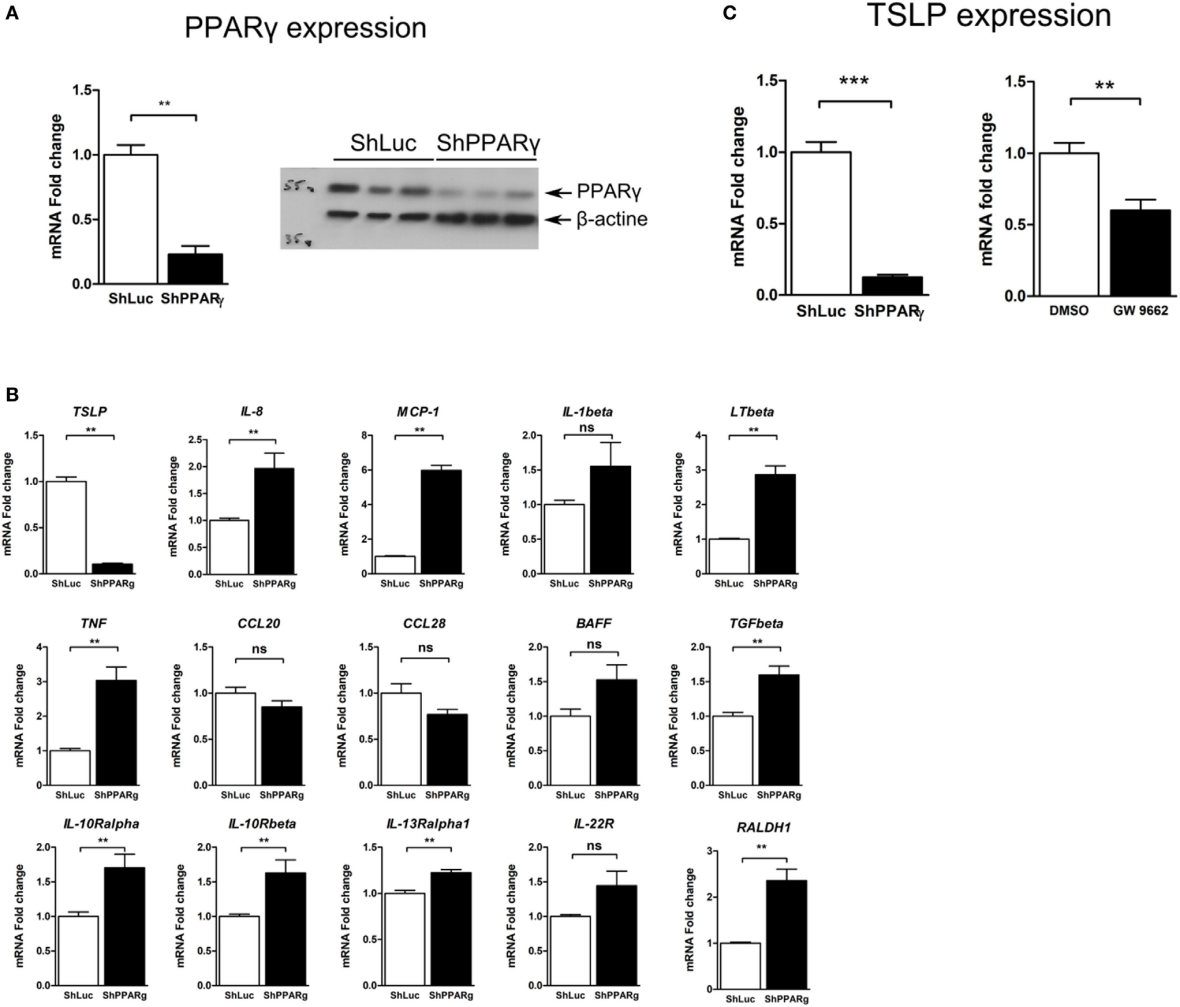
TSLP expression is strongly reduced in peroxisome proliferator activated receptor-gamma (PPARγ) knock-down Caco-2 cells. **(A)** Caco-2 colorectal cell line knock-down for PPARγ (ShPPARγ) expressed significantly fewer PPARγ transcripts and protein compared to ShLuc control cells. Quantitative expression of mRNA was assessed by quantitative PCR (qPCR). Results represent the mean values of sextuplicate ± SD of the fold change of PPARγ expression normalized to GAPDH level. The expression level measured in ShLuc cells (arbitrarily defined as one) was used as reference. Protein level was assessed by Western blot. The results represent a triplicate of the same clone of ShLuc and ShPPARγ Caco-2 cells, respectively. **(B)** Targeted approach in order to identify genes with different expression profile in PPARγ knock-down Caco-2 cells (ShPPARγ) compared to control cells (ShLuc)—Results of qPCR analysis. We selected various genes potentially expressed by intestinal epithelial cells and tested their expression in ShPPARγ/ShLuc Caco-2 cells. Results represent the mean ± SEM (2 independent experiments in triplicate) of the fold change of selected genes expression normalized to GAPDH level. The expression level measured in control cells was used as reference and defined as 1. **(C)** qPCR analysis of TSLP gene expression in PPARγ knock-down Caco-2 cells (ShPPARγ) compared to control cells (ShLuc). Results represent mean ± SEM (5 independent experiments in triplicate or sextuplicate, *n* = 18) of the fold change of TSLP gene expression normalized to GAPDH level. The expression level measured in control cells was used as reference and defined as 1. Caco-2 cells were stimulated for 24 h with GW9662. Results represent mean ± SEM (2 independent experiments in triplicate or sextuplicate, *n* = 9) of the fold change of TSLP gene expression normalized to GAPDH level. The expression level measured in control cells was used as reference and defined as 1. **P < 0.01; ***P < 0.001; ns, not significant. MCP-1, Monocyte chemoattractant protein-1; LTβ, Lymphotoxin-β; IL-1β, Interleukin-1 β; TSLP, thymic stromal lymphopoietin; TNF, tumor necrosis factor; CCL28, Chemokine (C-C motif) ligand 28; BAFF, B-cell activating factor; IL-10Rα, interleukin-10 receptor α chain; CCL20, Chemokine (C-C motif) ligand 20; TGFβ, Transforming growth factor β; RALDH-1, retinaldehyde dehydrogenase 1; IL-13Rα1, interleukin-13 receptor α1 chain; IL-22R, interleukin-22 receptor; IL-10Rβ, interleukin-10 receptor β chain.

In order to identify factors whose expression depends on PPARγ in the CEC and which might be involved in the immunoregulatory properties of these cells, we have used a targeted approach and first investigated the mRNA expression of some genes encoding cytokines and cytokine receptors in PPARγ knock-down colonic epithelial Caco-2 cells. To this end, we used a Caco-2 ShPPARγ cell line that stably expresses a short hairpin anti-sense RNA against PPARγ, leading to specific downregulation of PPARγ (10) (Figure 1A). Compared to control cells (Caco-2 ShLuc cells, expressing a control shRNA directed against the luciferase gene), the expression of PPARγ in Caco-2 ShPPARγ cell line was reduced approximately by 70% and this decrease was equivalent to those we observed in CEC from UC patients (9, 10).

Thus, using this system, we observed that the expression of some pro-inflammatory genes such as interleukin-1β or MCP-1 was significantly upregulated in Caco-2 ShPPARγ cells (Figure 1B). More remarkably, we observed a dramatic decrease of expression of the mRNA encoding TSLP cytokine (Figure 1B). To confirm this result, we measured the expression level of TSLP mRNA in independent experiments and systematically found that TSLP mRNA level was significantly reduced by about 90% in ShPPARγ Caco-2 cells compared to the ShLuc cells (Figure 1C). In order to exclude the possibility of off-target effects of anti-sense RNA, we investigated the effect of the PPARγ antagonist GW9662. We observed that GW9662 also significantly suppressed TSLP expression in Caco-2 cells (Figure 1C). These first results demonstrate that TSLP expression is abrogated in Caco-2 cells when PPARγ is inhibited.

### Activation of PPARγ-Induced TSLP Expression

**Figure 2:**
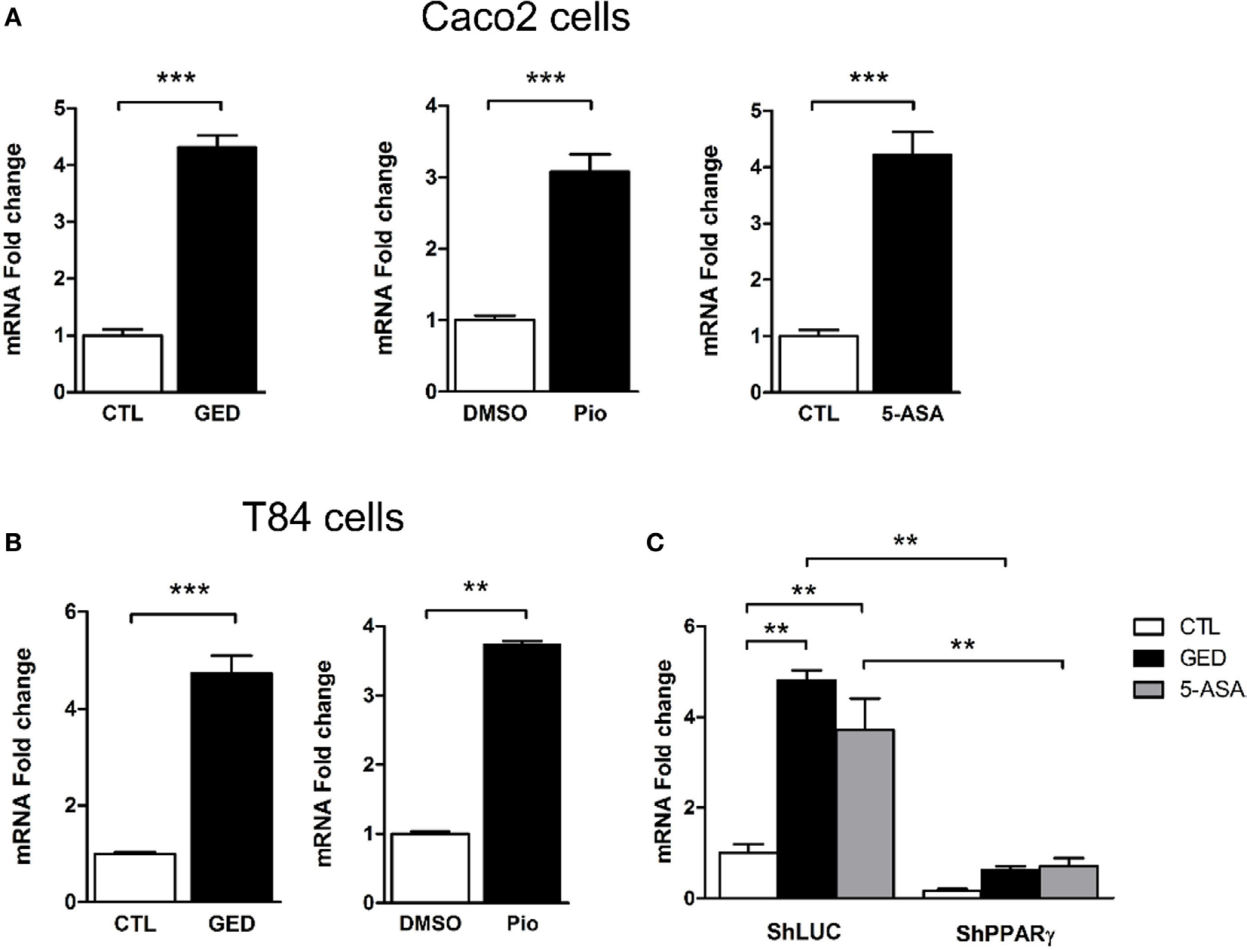
Activation of peroxisome proliferator activated receptor-gamma (PPARγ) in IEC cell lines induced TSLP expression. **(A)** qPCR analysis of TSLP gene expression in stimulated Caco-2 cells. Cells were stimulated for 24 h with each agonist. Results represent mean ± SEM (2 independent experiments in triplicate or sextuplicate, 9 < *n* < 12) of the fold change of TSLP gene expression normalized to GAPDH level. The expression level measured in control cells was used as reference and defined as 1. **(B)** qPCR analysis of TSLP gene expression in stimulated T84 cells. Cells were stimulated for 24 h with each agonist. Results represent mean ± SEM (2 independent experiments in triplicate or sextuplicate, 9 < *n* < 12) of the fold change of TSLP gene expression normalized to GAPDH level. The expression level measured in control cells was used as reference and defined as 1. **(C)** qPCR analysis of TSLP gene expression in ShPPARγ cells compared to ShLuc cells stimulated by GED and 5-ASA. Results represent mean ± SEM (two independent experiments in triplicate, *n* = 6) of the fold change of TSLP gene expression normalized to GAPDH level. The expression level measured in control cells (unstimulated ShLuc cells) was used as reference and defined as 1. **P < 0.01; ***P < 0.001.

To further explore the regulation of TSLP expression by PPARγ in CECs, we measured TSLP mRNA expression after stimulation of Caco-2 cells with 3 different PPARγ agonists: pioglitazone (Pio; 10 µM), a well characterized PPARγ ligand belonging to the anti-diabetic thiazolidinedione drug class (22), 5-amino salicylic acid (5-ASA; 30 mM) (23), and a new PPARγ modulator, we developed and named GED-0507-34-Levo (GED; 30 mM) (24). All PPARγ ligands tested were able to significantly induce the expression of TSLP mRNA in Caco-2 cells (Figure 2A). These results were confirmed in another IEC line (T84), which expresses both TSLP and PPARγ (Figure 2B). In order to confirm the involvement of PPARγ in this process, we next assessed the induction of TSLP mRNA expression by PPARγ ligands in PPARγ knock-down Caco-2 cells. We first observed that 5-ASA and GED significantly induce TSLP mRNA expression in Caco-2 ShLuc control cells in an identical manner than in normal Caco-2 cells (Figure 2C), while in ShPPARγ Caco-2 cells, both 5-ASA and GED-dependent expression of TSLP mRNA were strongly reduced (Figure 2C). This result shows that PPARγ potentiates *tslp* expression in Caco-2 cells. Taken together, our results support the hypothesis that PPARγ is a key factor controlling TSLP expression in CEC.

### PPARγ Regulates the Expression of the sfTSLP

**Figure 3:**
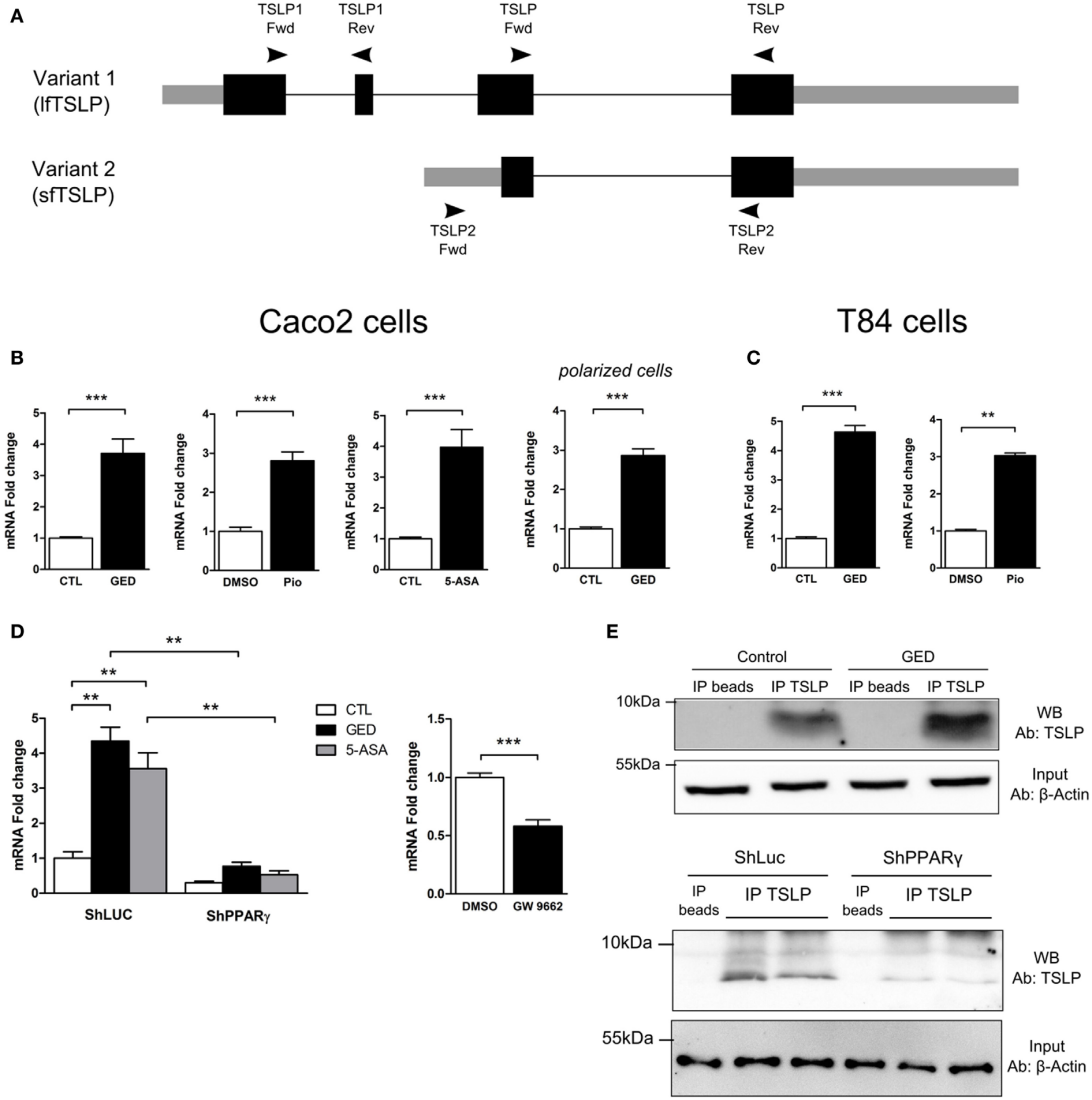
Activation of peroxisome proliferator activated receptor-gamma (PPARγ) in IEC cell lines induced the expression of the short isoform of TSLP (sfTSLP). **(A)** Schematic representation of the structural organization of the TSLP gene with its two isoforms. Variant 1 represents the long form TSLP (lfTSLP) and variant 2 represents the short isoform (sfTSLP). The rectangles represent the exons, the gray parts corresponding to the untranslated regions and the black parts corresponding to the translated regions. The oligonucleotides used to amplify the TSLP gene are depicted. Oligonucleotides TSLP Fwd/TSLP Rev do not discriminate between both isoforms. Oligonucleotides TSLP1 Fwd/TSLP1 Rev specifically amplify lfTSLP and oligonucleotides TSLP2 Fwd/TSLP2 Rev specifically amplify sfTSLP. **(B)** qPCR analysis of sfTSLP gene expression in stimulated Caco-2 cells. Cells were stimulated for 24 h with each agonist. Results represent mean ± SEM (2 independent experiments in triplicate or sextuplicate, 9 < *n* < 12) of the fold change of TSLP gene expression normalized to GAPDH level. The expression level measured in control cells was used as reference and defined as 1. **(C)** qPCR analysis of sfTSLP gene expression in stimulated T84 cells. Cells were stimulated for 24 h with each agonist. Results represent mean ± SEM (2 independent experiments in triplicate or sextuplicate, 6 < *n* < 12) of the fold change of sfTSLP gene expression normalized to GAPDH level. The expression level measured in control cells was used as reference and defined as 1. **(D)** qPCR analysis of sfTSLP gene expression in Caco-2 ShPPARγ cells compared to Caco-2 ShLuc cells stimulated by GED and 5-ASA. Results represent mean ± SEM (two independent experiments in triplicate, *n* = 6) of the fold change of sfTSLP gene expression normalized to GAPDH level. The expression level measured in control cells (unstimulated ShLuc cells) was used as reference and defined as 1. Caco-2 cells were stimulated for 24 h with GW9662. Results represent mean ± SEM (two independent experiments in triplicate or sextuplicate, *n* = 9) of the fold change of sfTSLP gene expression normalized to GAPDH level. The expression level measured in control cells was used as reference and defined as 1. **(E)** sfTSLP protein expression in Caco-2 stimulated with GED and in Caco-2 ShLuc/ShPPARγ. Protein extracts were first subjected to an immunoprecipitation step with an antibody directed against TSLP (IP TSLP) or without any antibody (IP beads). sfTSLP protein was detected by western-blot in the immunoprecipitated materials. The detection of β-actin protein in the input (1% input) before immunoprecipitation was used as loading control. **P < 0.01; ***P < 0.001.

Three distinct mRNA variants can be transcribed from the human *tslp* gene, but only two encode functional protein. Variant 1 is composed of 4 exons which encode the long form TSLP (lfTSLP) of 159 amino acids length. Variant 2 is composed of 2 exons which encode the sfTSLP consisting in the 63 and/or 60 C terminal amino acids of long TSLP (Figure 3A). It is only very recently that studies have been interested in distinguishing these 2 isoforms and their possible distinct functions (25–27). It appears that lfTSLP mRNA and protein are upregulated in response to inflammatory stimuli and therefore associated to inflammatory conditions such as asthma, dermatitis, or IBD (26). Conversely, sfTSLP is constitutively expressed in healthy mucosa of the digestive tract as well as in the skin (25, 26). The cues that drive this distinct expression pattern are unknown. To test the implication of PPARγ, we designed isoform-specific primers (Figure 3A; Table S1 in Supplementary Material for primer sequences) and assessed the transcription of the two TSLP variants. lfTSLP transcript was amplified from bronchial epithelial cells (H292) stimulated with polyinosinic-polycytidylic acid (polyI:C) (Figure S1 in Supplementary Material), but no lfTSLP transcript was detected in our quantitative RT-PCR experiments in Caco-2 and T84 cells, both at the basal level nor after stimulation with PPARγ agonists. On the other hand, the three PPARγ agonists were all able to significantly induce sfTSLP mRNA expression, both in Caco-2 and T84 cells (Figures 3B,C). The Caco-2 cells have the ability to spontaneously differentiate (when reaching confluence) into polarized IECs which express, for instance, tight junction proteins and develop transepithelial electric resistance. In order to test whether the induction of sfTSLP by PPARγ agonist was maintained or modified by Caco-2 cells differentiation, we also assessed the induction of sfTSLP by GED in polarized Caco-2 cells. To this end, Caco-2 cells were grown on transwell filters during 21 days to allow cell polarization, and then cells were stimulated with PPARγ agonist for 24 h. In these conditions, activation of PPARγ with GED still resulted in a significant three-fold induction of sfTSLP mRNA expression (Figure 3B). We next investigated the stimulation of sfTSLP mRNA expression by PPARγ ligands in PPARγ knock-down Caco-2 cells. We found that 5-ASA and GED significantly induce sfTSLP mRNA expression in Caco-2 ShLuc control cells (to the same extend to what we observed in Figure 2C with non-isoform specific primers), while in ShPPARγ Caco-2 cells, both 5-ASA and GED-dependent expression of sfTSLP mRNA were strongly compromised (Figure 3D). Treatment of Caco-2 cells with the PPARγ antagonist GW9662, confirmed that blocking PPARγ activity significantly impact the expression of sfTSLP (Figure 3D). Finally, we tried to confirm these results by detecting and evaluating sfTSLP protein level in stimulated Caco-2 cells. Given that the amino acid sequence of sfTSLP perfectly overlap the C-terminal sequence of lfTSLP, it is not possible to have an antibody specifically directed against sfTSLP. However, by using an antibody raised against the C-terminal part common to both proteins, it should be possible to distinguish the two isoforms in western-blot analysis due to their different molecular weights [apparent molecular weight of 23 kDa for lfTSLP and 9 kDa for sfTSLP (25)]. Nevertheless, we never succeeded to detect sfTSLP in protein extracts from Caco-2 cells (stimulated or not) directly in western-blot analysis (not shown). We hypothesized that it could be due to a low level of sfTSLP protein expression and, thus, in order to increase TSLP protein concentration, we first performed an immunoprecipitation step before western-blot analysis. This approach allowed us to detect sfTSLP protein in Caco-2 stimulated with GED as well as in Caco-2 ShLuc/ShPPARγ (Figure 3E). No signal was observed in the lanes corresponding to the immunoprecipitation control (Figure 3E “IP beads,” which correspond to the immunoprecipitation of protein extracts without any anti-TSLP antibody), whereas, we detected a band migrating just below 10 kDa in the lane corresponding to the proteins immunoprecipitated with anti-TSLP antibody (Figure 3E, “IP TSLP”). We concluded that this band corresponds to sfTSLP protein. Moreover, we observed a stronger signal in the lane corresponding to proteins immunoprecipitated from Caco-2 cells stimulated with GED (Figure 3E, GED lane “IP TSLP”) compared to unstimulated Caco-2 cells (Figure 3E, control lane “IP TSLP”) and fewer sfTSLP were immunoprecipitated from ShPPARγ Caco-2 cells than from ShLuc Caco-2 cells (Figure 3E, ShPPARγ “IP TSLP” lanes compared to ShLuc “IP TSLP” lanes). Given that equal amount of protein was engaged in each immunoprecipitation, we concluded from this experiment that modulation of PPARγ in Caco-2 cells impacts sfTSLP protein expression. Taken together, the results of this part strongly suggest that PPARγ is an important transcription factor controlling sfTSLP expression in CEC.

### PPARγ Functionally Binds to the Promoter of sfTSLP Gene

**Figure 4:**
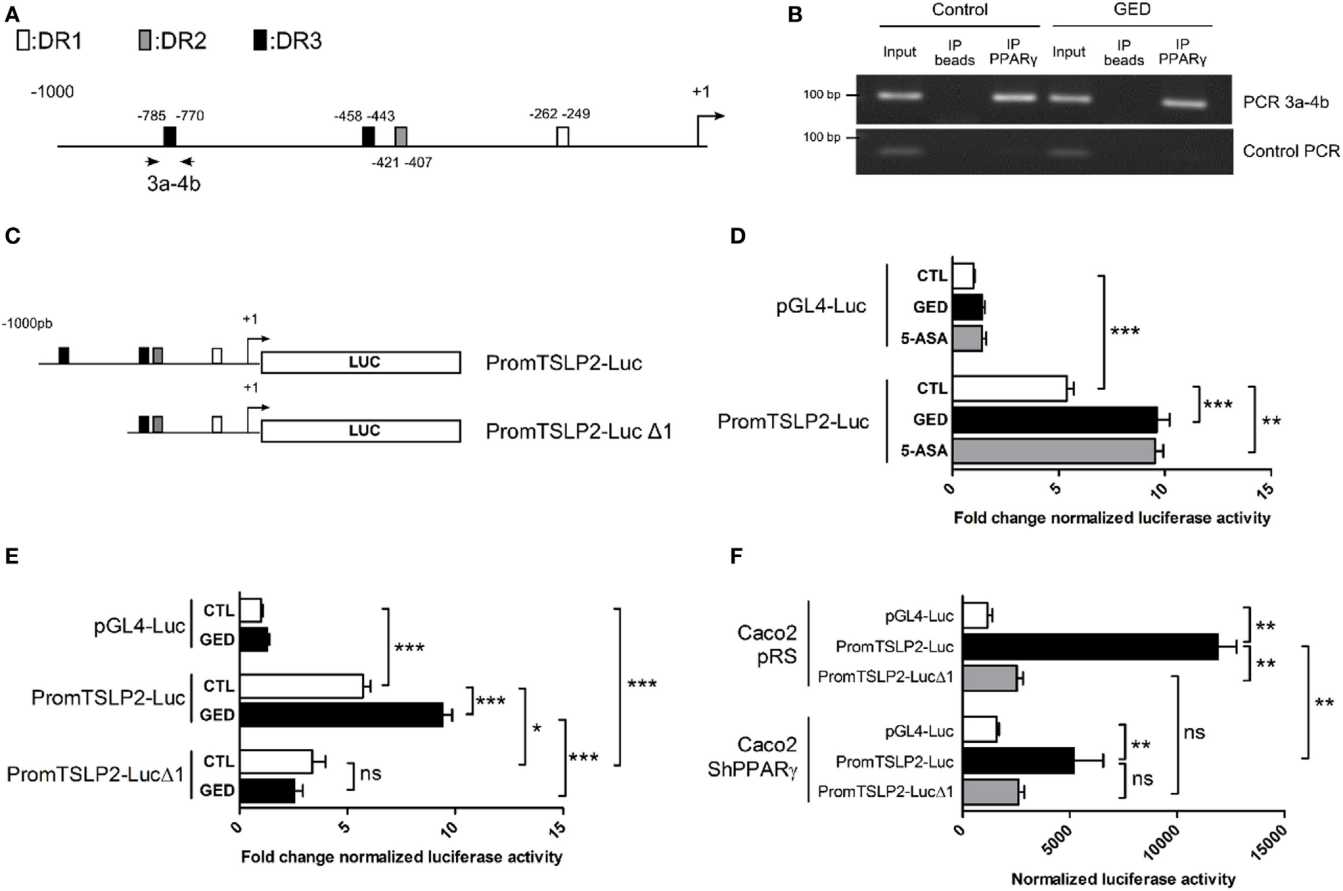
Peroxisome proliferator activated receptor-gamma (PPARγ) binds functionally to the promoter of sfTSLP gene. **(A)** Schematic representation of the promoter of the sfTSLP gene (1,000 base pairs upstream of the transcription start site) showing the potential presence of PPAR response element predicted by *in silico*. **(B)** Picture showing PCR amplification of the 3a–4b fragment in ChIP assay from Caco-2 cells. “Control PCR” corresponds to the amplification of a genomic region unrelated to sfTSLP promoter and serves as a control of specificity for the ChIP analysis. **(C)** Schematic representation of the two reporter constructions used in transient transfection and reporter gene assays. **(D,E)** Luciferase gene reporter assays in Caco-2 cells transfected with PromTSLP2-Luc and PromTSLP2-LucΔ1 reporter constructs. Cells transfected with empty pGL4Luc plasmid were used as control. Results represent the mean ± SEM (3 independent experiments in triplicate or sextuplicate, 9 < *n* < 15) of the fold change of luciferase activity normalized for protein content. The normalized Luc activity obtained in unstimulated Caco-2 cells transfected with empty pGL4Luc was used as reference and defined as 1. **(F)** Luciferase gene reporter assays in in PPARγ knock-down Caco-2 cells transfected with PromTSLP2-Luc and PromTSLP2-LucΔ1 reporter constructs. Cells transfected with empty pGL4Luc plasmid were used as control. Results represent the mean ± SEM of luciferase activity normalized for protein content (two independent experiments in triplicate). *P < 0.05; **P < 0.01; ***P < 0.001; ns, not significant.

To further confirm the regulation of sfTSLP expression by PPARγ in CECs, we next investigated the presence of PPAR response element (PPRE) sequences in the sfTSLP gene promoter. *In silico* analysis of the 1,000 base pairs (bp) upstream from the transcription start site of the human sfTSLP gene revealed the presence of several potential PPRE (DR1, DR2, and DR3) by which PPARγ may regulate sfTSLP gene expression (Figure 4A; Figure S2 in Supplementary Material). Chromatin immunoprecipitation analysis of these putative PPRE revealed notably that a DR3 located between −785 (bp) and −770 bp upstream of the transcription start site was bound by PPARγ within the sfTSLP gene promoter in Caco-2 cells (both in control and stimulated cells) (Figure 4B). In order to test whether this binding was functional, we constructed two reporter plasmids: in the first one, the genomic fragment corresponding to the 1,000 bp upstream from the transcription start site of the human sfTSLP gene was cloned upstream to the luciferase reporter gene into a pGL4 vector (promTSLP2-Luc construct; Figure 4C). In the second one, the genomic region containing the putative PPRE DR3 bound by PPARγ was deleted, giving “promTSLP2-Luc Δ1” construct (Figure 4C). Then, both plasmids were tested in transient reporter gene assays in Caco-2 cells (Figures 4D–F). First, in unstimulated conditions, the transfection of promTSLP2-Luc plasmids led to a significant five fold increase of luciferase activity compared to the transfection of empty pGL4-Luc vector, indicating that the portion of promoter present in the promTSLP2-Luc construct is active in Caco-2 cells (Figure 4D, compare white bar graphs). Stimulation of “promTSLP2-Luc”-transfected cells with GED or 5-ASA induced a significant additional increase of the reporter gene activity compared to unstimulated transfected cells, suggesting that putative functional PPARγ-responsive sequences are present within the genomic fragment cloned in promTSLP2-Luc construct (Figure 4D, compare white, black, and gray bar graphs in promTSLP2-Luc transfected cells). In order to test whether the DR3 identified by the ChIP experiments could be this functional element, we tested the “promTSLP2-Luc Δ1” reporter construct in transient transfection (Figure 4E). As expected, transfection of Caco-2 cells with “promTSLP2-Luc” contruct gave the same luciferase induction as previously observed and, while in unstimulated conditions, the construction “promTSLP2-Luc Δ1” retained some transcriptional activity (Figure 4E, compare white bar graphs), the inducing effect of the GED was totally lost (Figure 4E, compare white and black bar graphs in promTSLP2-Luc Δ1 transfected cells). This result strongly suggests that the DR3 found to be bound by PPARγ in ChIP experiment is functional in the sfTSLP gene promoter. To irrevocably establish the involvement of PPARγ in the regulation of the activity of the sfTSLP promoter, we transfected PPARγ knock-down Caco-2 cells with our two reporter plasmids (Figure 4F). In control cells (Caco-2 pRS cells, corresponding to Caco-2 cells having stably integrated the empty pRS vector used to express shRNA), the transfection of “promTSLP2-Luc” plasmid led to a significant increase of luciferase activity compared to cells transfected with the empty pGL4-Luc vector. The transfection of “promTSLP2-Luc Δ1” provoked a dramatic decrease of luciferase activity compared to cells transfected with the “promTSLP2-Luc” plasmid, confirming our last result and the importance of the genomic region encompassing the DR3 element in the transcriptional activity of the sfTSLP gene promoter. The knock-down of PPARγ expression in Caco-2 shPPARγ cells significantly decreased the luciferase activity of the “promTSLP2-Luc” construction (Figure 4F, compare black bar graphs), while it had no effect on the remaining transcriptional activity of the “promTSLP2-Luc Δ1” construction (Figure 4F, compare gray bar graphs). These results clearly confirmed that PPARγ is involved in the control of the activity of the sfTSLP promoter. Taking together, our results demonstrate that PPARγ binds functionally to the promoter of sfTSLP and show that PPARγ is a transcriptional regulator of sfTSLP gene.

### Decreased sfTSLP Expression in Patients with UC Correlates with PPARγ Expression

**Figure 5:**
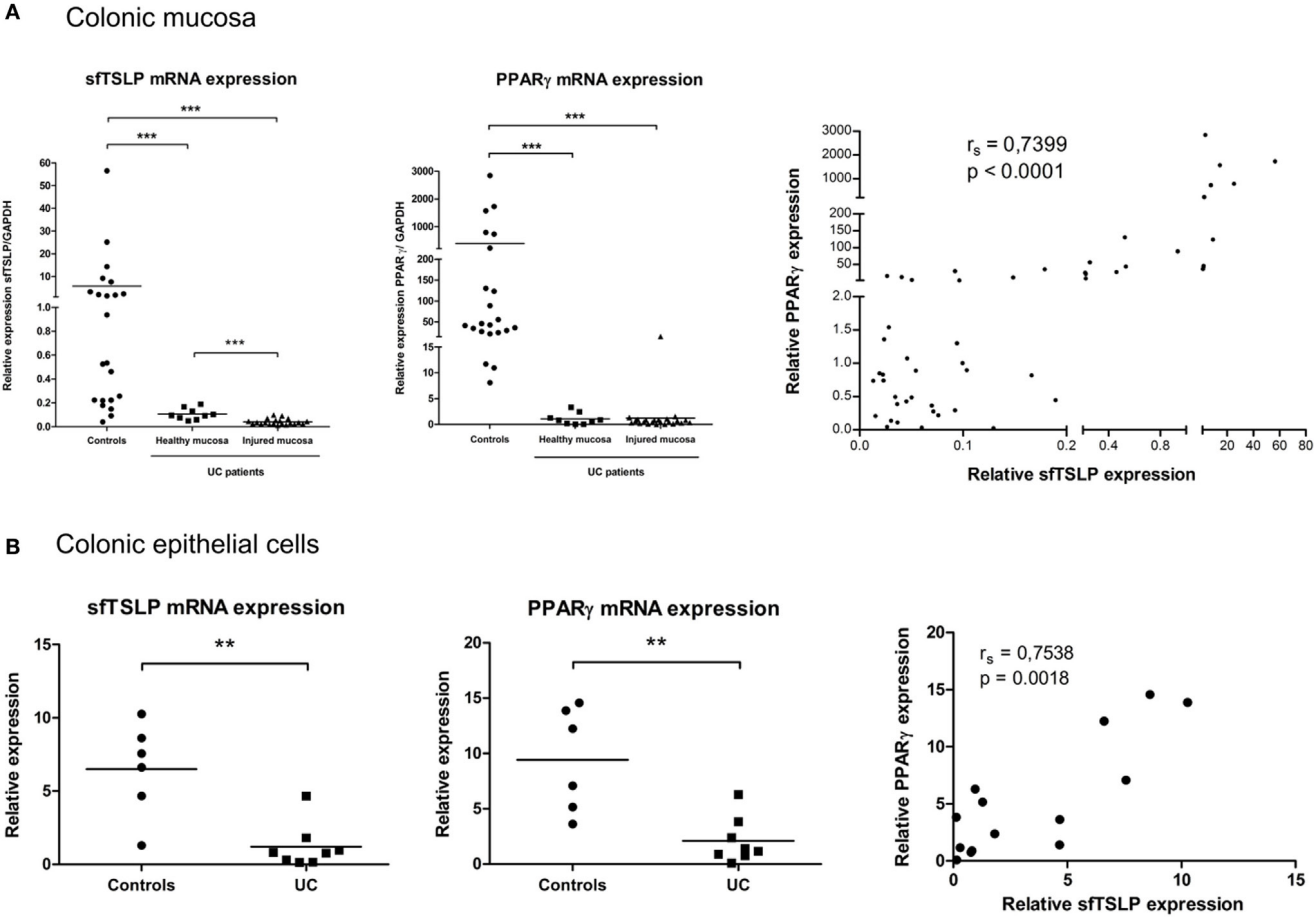
sfTSLP expression is decreased in ulcerative colitis (UC) patients and correlates with peroxisome proliferator activated receptor-gamma (PPARγ) expression level. **(A)** Colonic mucosa were obtained from control subjects (*n* = 22) or patients with UC (healthy mucosa, *n* = 9; injured mucosa, *n* = 21). Quantitative expression of PPARγ and sfTSLP mRNA were assessed by qPCR and normalized to GAPDH level. Transcript levels of sfTSLP in colonic mucosa from both controls and UC patients were correlated with PPARγ mRNA level. *P*-value was determined by the non-parametric Spearman test. **(B)** Colonic epithelial cells (CEC) were purified from resected colon specimens of control subjects (*n* = 6) or untreated patients with UC (*n* = 8). Quantitative expression of PPARγ and sfTSLP mRNA were assessed by qPCR and normalized to GAPDH level. Transcript levels of sfTSLP in CEC from both controls and UC patients were correlated with PPARγ mRNA level. *P*-value was determined by the non-parametric Spearman test. Horizontal bar indicates the mean value. **P < 0.01, ***P < 0.001.

We and others have previously demonstrated that PPARγ expression is impaired in the colonic mucosa and in the CEC of patients suffering from UC (9, 10, 28). Given our results demonstrating the relationship between PPARγ and sfTSLP, we next assessed the expression level of TSLP isoforms in the colonic mucosa and CEC of UC patients and non-IBD controls. We first confirmed the significant impairment of PPARγ mRNA expression, both in healthy and injured colonic mucosa from UC patients compared to control specimens (Figure 5A). We never obtained satisfactory lfTSLP mRNA detection in our colonic patient samples, even when trying with different combinations of newly designed and already published PCR primers (26) (see Figure S1 in Supplementary Material). Interestingly, we observed that, in UC patients, the expression of sfTSLP was significantly decreased in healthy and injured mucosa compared to controls specimens (Figure 5A), and we found a significant correlation between the expression level of PPARγ and sfTSLP in the colonic mucosa of all our patient samples (UC and controls) (Figure 5A). We finally assessed the expression levels of sfTSLP and PPARγ in CEC and found similar results: sfTSLP mRNA expression level was significantly decreased specifically in purified CEC from patients with UC and correlated with PPARγ mRNA expression level (Figure 5B). Taken together, these results therefore strengthen the relationship between PPARγ and sfTSLP and support the hypothesis that, in UC, the impaired expression of PPARγ in colonic mucosa and CEC could play a role in the loss of sfTSLP expression.

## Discussion

To our knowledge, the present study is the first to address the transcriptional regulation of the sfTSLP, at least in the intestine. Our results identified the nuclear receptor PPARγ as an important transcriptional regulator of sfTSLP in CEC and strongly suggest that the lack of expression of sfTSLP that we observed in the colonic mucosa and CEC of patients with UC is related to that of PPARγ.

The distinction between the two isoforms of TSLP has only been made since very recently. It is now a fundamental aspect of the biology of this cytokine because the two isoforms possess distinct functions and expression profiles (26, 27, 29). *In vitro*, lfTSLP expression is regulated by inflammatory stimuli. For instance, only the expression of lfTSLP is induced in normal human bronchial epithelial cells after polyI:C exposure (30). In human keratinocytes, toll-like receptors ligands (polyI:C, FSL-1, and flagellin) as well as pro-inflammatory cytokines (IFNγ, TNF, IL-1β) are potent inducers of lfTSLP, but not of sfTSLP (25, 31). Finally, exposure of Caco-2 cells to *Salmonella typhimurium* induces upregulation of lfTSLP protein expression concomitantly to a downregulation of sfTSLP expression (26). Consistent with these *in vitro* observations, lfTSLP was found to be upregulated *in vivo* in inflammatory conditions such as IBD, asthma or atopic dermatitis and NF-κB was identified as a critical factor for inflammation-induced expression of lfTSLP (32, 33). In terms of function, although most of the work done so far has very rarely distinguished the two isoforms, the data suggest that lfTSLP has proinflammatory properties (27, 29). Conversely, anti-inflammatory properties have been suggested, both *in vitro* and *in vivo*, for sfTSLP which is the predominant form of TSLP, constitutively expressed at steady state (26, 29). More interestingly, recent work has demonstrated that synthetic sfTSLP displays antimicrobial activity, suggesting that this isoform may act as an antimicrobial peptide (25, 27). In this context, our results appear to be highly relevant. Indeed, although the exact role of PPARγ in the intestinal tract still remains to be characterized, it has been suggested that this factor plays a key role in gut homeostasis. Depletion of PPARγ in mouse CEC increases susceptibility to experimental colitis, while activation of this receptor reduces intestinal inflammation and disease severity both in experimental models of colitis and in UC patients. One explanation of this positive role on gut homeostasis is the potential PPARγ-dependent antimicrobial barrier function. Indeed, it has been suggested that PPARγ mediates antimicrobial immunity in the colon, notably trough the control of antimicrobial β-defensin-1 and mannose-binding lectin gene expression (34, 35). Therefore, the identification of the antimicrobial peptide sfTSLP as new PPARγ-target gene in CEC broadens the role of this nuclear receptor both as an important antibacterial factor at the gut mucosal surfaces as well as a key actor during intestinal homeostasis.

To date, only one study has investigated the differential expression of the TSLP isoforms in the intestine under normal conditions and in patients suffering from IBD (26). One of the main finding of this work was that the sfTSLP/lfTSLP ratio was altered (decreased) in patients with UC due to an upregulation of lfTSLP expression in colonic mucosa from UC patients compared to control subjects, whereas sfTSLP levels were unchanged. This perturbation could reflect the loss of intestinal homeostasis and could lead (or participate) to an uncontrolled inflammatory response. However, contrary to the study of Fornasa et al., we did not detect lfTSLP mRNA, either in Caco-2 or T84 cells nor in our human colonic samples (both from control and UC patients). We used different primer pairs (our own primers and already published primers) and, although they all allowed amplifying the long form from H292 bronchial epithelial cells, no primer pair efficiently amplified lfTSLP from CEC or colonic mucosa. Despite this, we do believe that our results obtained on patient samples are not necessarily in conflict with those of Fornasa et al. (26), but could be rather in the same line. The decrease in expression of sfTSLP that we observed, in association with an apparent unmodified level of lfTSLP, results in a decrease in the sfTSLP/lfTSLP ratio as described in the work of Fornasa et al. On the other hand, the impaired expression of sfTSLP in the colonic mucosa of our patients with UC is consistent with the suggested immunoregulatory/antimicrobial role of this isoform. This decreased pattern of expression of sfTSLP in UC patients in our samples was confirmed by using the primer pair used to amplify sfTSLP by Fornasa et al. (Figure S3 in Supplementary Material). *In vitro*, it has been suggested that the expression of the short form is inhibited by proinflammatory stimuli (26). Because we also observed lowered sfTSLP expression in healthy mucosa from UC patients, it seems unlikely that the perturbed levels of sfTSLP are related to the inflammatory process in our samples. In addition, we did not observe any correlation between the expression levels of sfTSLP and IL-8 (Figure S4 in Supplementary Material). The significant correlation obtained between the expression levels of PPARγ and sfTSLP rather suggests that the defect of sfTSLP is related to the loss of expression of PPARγ typically observed in the CEC of UC patients. This correlation strengthens the functional link between these two factors. Moreover, these defects observed in inflamed and healthy colons of patients with UC could suggest a primary role of colonic PPARγ and sfTSLP impairment in the pathophysiology of UC. The discrepancy between the results published by Fornasa et al. and those of our study could be due to the nature of the colonic samples used. In the Fornasa study, it seems that most of the samples of UC patients came from the sigmoid colon, whereas in our work, very few samples are derived from sigmoidectomy but are rather more proximal colonic samples coming from partial or total colectomies (Table S2 in Supplementary Material). The expression profile of TSLP isoforms along the gut (proximal colon versus distal colon for example) is unknown, but it may exist some differences that could explain these divergent results. Thus, it would be important to precisely determine the expression profile of TSLP isoforms along the normal human gastrointestinal tract in order to better understand the biology of this cytokine.

It is important to note that the sfTSLP does not exist in mouse and has been isolated only in humans. Consequently, the relevance of using mice models to study the sfTSLP functions is limited. It has been suggested that intraperitoneal injection of sfTSLP slightly improves DSS-induced colitis, but the underlying mechanism was not investigated and thus remains unknown (26). Given the unique presence of sfTSLP in humans, it would be more relevant to test the antimicrobial activity of sfTSLP on commensal and pathological bacteria associated with human gut. The recent development of colonic organoids of human origin (36–38) should also make it possible to better understand the role of sfTSLP during intestinal homeostasis.

In conclusion, the present study has identified a new PPARγ target gene in CEC, confirming the key role of this nuclear receptor in antimicrobial immunity and during intestinal homeostasis. In addition, it suggests a new molecular mechanism involved in the loss of intestinal tolerance observed during UC.

## Ethics Statement

A local ethics committee (Comité de Protection des Personnes Nord Ouest IV, CHRU Lille, France) approved the study and all subjects gave informed consent (No. DC-2008-642). Surgical colonic samples were obtained from patients with an established diagnosis of UC according to international criteria and from control patients (patients underwent surgery for colorectal cancer and patients for diverticulitis of the sigmoid). The detailed characteristics of patient cohort are given in Table S2 in Supplementary Material.

## Author Contributions

AMM, PD, LD, and BB designed the study. AMM, AL, SS, and BB performed experiments and analysis of the data. LS collected human colonic samples and managed clinical data. AMM, LD, and BB wrote the paper and participated to the critical reading of the manuscript.

## Conflict of Interest Statement

The authors declare that the research was conducted in the absence of any commercial or financial relationships that could be construed as a potential conflict of interest.

## Footnotes

^1^http://primerdepot.nci.nih.gov.

^2^http://www.nubiscan.unibas.ch/.

^3^http://www.classicrus.com/PPRE/.
