# Supplementary material for: The Expression of the Short Isoform of Thymic Stromal Lymphopoietin in the Colon Is Regulated by the Nuclear Receptor Peroxisome Proliferator Activated Receptor-Gamma and Is Impaired during Ulcerative Colitis
Source: Front Immunol. 2017 Sep 4;8:1052. doi: 10.3389/fimmu.2017.01052 (PMC5591373; doi:10.3389/fimmu.2017.01052)
Supplement: Supplementary file 1 [file Data_Sheet_1.PDF]

SUPPLEMENTARY FIGURE S1

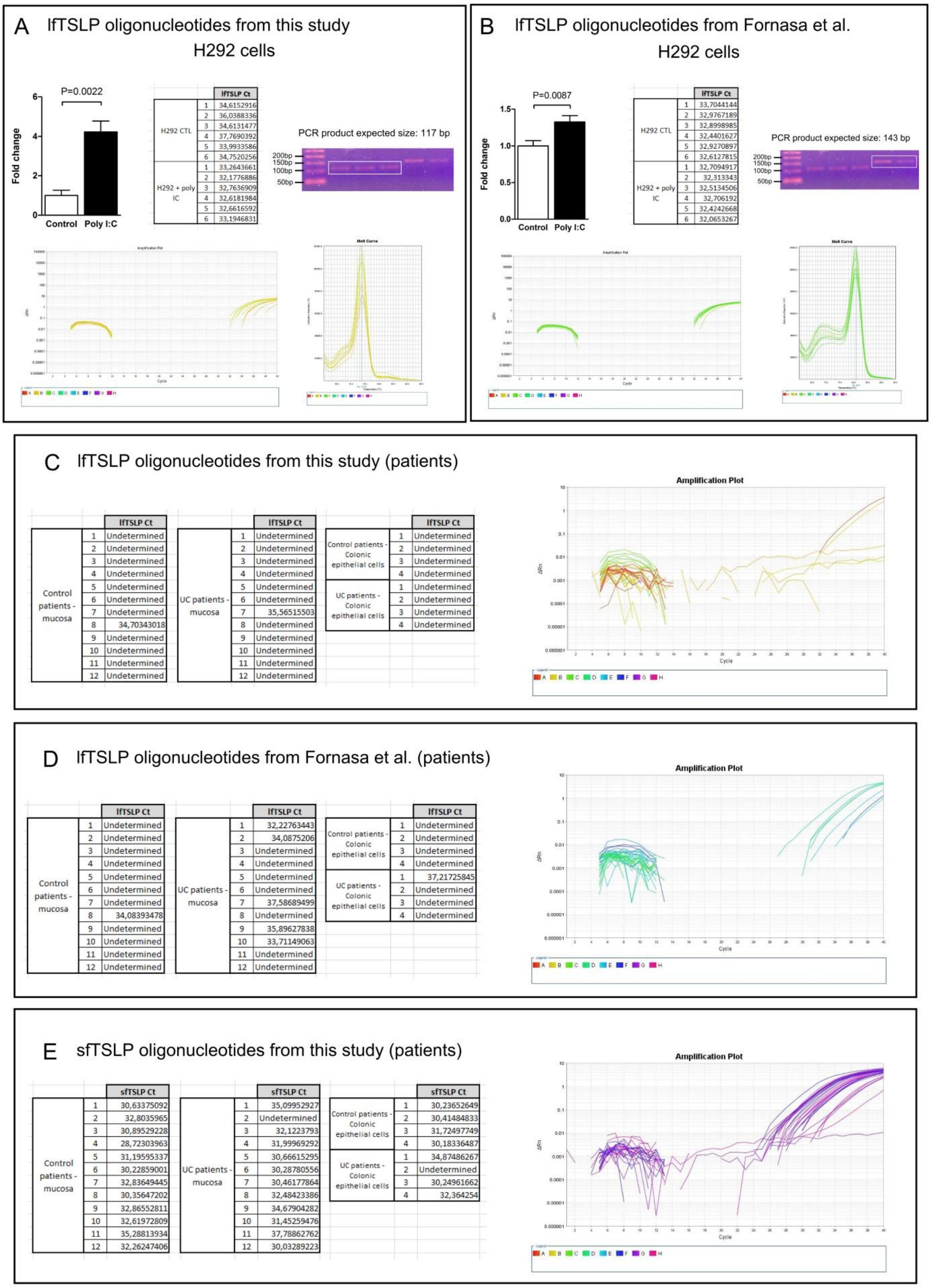

A

		lfTSLP Ct
H292 CTL	1	34,6152916
	2	36,0388336
	3	34,6131477
	4	37,7690392
	5	33,9933586
	6	34,7520256
H292 + poly IC	1	33,2643661
	2	32,1776886
	3	32,7636909
	4	32,6181984
	5	32,6616592
	6	33,1946831

B

		lfTSLP Ct
H292 CTL	1	33,7044144
	2	32,9767189
	3	32,8998985
	4	32,4401627
	5	32,9270897
	6	32,6127815
H292 + poly IC	1	32,7094917
	2	32,313343
	3	32,5134506
	4	32,706192
	5	32,4242668
	6	32,0653267

C

		lfTSLP Ct
Control patients - mucosa	1	Undetermined
	2	Undetermined
	3	Undetermined
	4	Undetermined
	5	Undetermined
	6	Undetermined
	7	Undetermined
	8	34,70343018
	9	Undetermined
	10	Undetermined
	11	Undetermined
	12	Undetermined

		lfTSLP Ct
UC patients - mucosa	1	Undetermined
	2	Undetermined
	3	Undetermined
	4	Undetermined
	5	Undetermined
	6	Undetermined
	7	35,56515503
	8	Undetermined
	9	Undetermined
	10	Undetermined
	11	Undetermined
	12	Undetermined

		lfTSLP Ct
Control patients - Colonic epithelial cells	1	Undetermined
	2	Undetermined
	3	Undetermined
	4	Undetermined
UC patients - Colonic epithelial cells	1	Undetermined
	2	Undetermined
	3	Undetermined
	4	Undetermined

D

		lfTSLP Ct
Control patients - mucosa	1	Undetermined
	2	Undetermined
	3	Undetermined
	4	Undetermined
	5	Undetermined
	6	Undetermined
	7	Undetermined
	8	34,08393478
	9	Undetermined
	10	Undetermined
	11	Undetermined
	12	Undetermined

		lfTSLP Ct
UC patients - mucosa	1	32,22763443
	2	34,0875206
	3	Undetermined
	4	Undetermined
	5	Undetermined
	6	Undetermined
	7	37,58689499
	8	Undetermined
	9	35,89627838
	10	33,71149063
	11	Undetermined
	12	Undetermined

		lfTSLP Ct
Control patients - Colonic epithelial cells	1	Undetermined
	2	Undetermined
	3	Undetermined
	4	Undetermined
UC patients - Colonic epithelial cells	1	37,21725845
	2	Undetermined
	3	Undetermined
	4	Undetermined

E

		sfTSLP Ct
Control patients - mucosa	1	30,63375092
	2	32,8035965
	3	30,89529228
	4	28,72303963
	5	31,19595337
	6	30,22859001
	7	32,83649445
	8	30,35647202
	9	32,86552811
	10	32,61972809
	11	35,28813934
	12	32,26247406

		sfTSLP Ct
UC patients - mucosa	1	35,09952927
	2	Undetermined
	3	32,1223793
	4	31,99969292
	5	30,66615295
	6	30,28780556
	7	30,46177864
	8	32,48423386
	9	34,67904282
	10	31,45259476
	11	37,78862762
	12	30,03289223

		sfTSLP Ct
Control patients - Colonic epithelial cells	1	30,23652649
	2	30,41484833
	3	31,72497749
	4	30,18336487
UC patients - Colonic epithelial cells	1	34,87486267
	2	Undetermined
	3	30,24961662
	4	32,364254

Supplementary figure S1: lfTSLP expression is detected and induced by polyinosinic-polycytidylic acid (polyI:C) in H292 bronchial epithelial cells but is mostly undetectable in our patients' samples.

(A) and (B): qPCR analysis of lfTSLP gene expression in H292 cells stimulated with poly I:C for 8 h. **Panel A** presents the results obtained with a primer pair designed by our own for this study. **Panel B** presents the results obtained with a primer pair described in (Fornasa et al., 2015). The data correspond to amplification data obtained from samples retro-transcribed at the same time, in the same conditions and ran on the same qPCR plaque.

In each panel:

- Bar graph result represents mean ± SEM (2 independent experiments in triplicate, n=6) of the fold change of TSLP gene expression (mRNA level) normalized to GAPDH level. P value (two-tailed nonparametric Mann-Whitney test) is indicated.
- The chart presents the raw data of Ct values for lfTSLP amplification.
- The amplification and melt curves obtained for each primer pair
- Agarose gel stained with ethidium bromide showing the PCR product obtained for lfTSLP. PCR product migrates to the expected size (117 bp and 143 bp respectively).

(C) Amplification of lfTSLP isoform from patients' samples using our own primer pair. The chart presents the raw data of Ct values for lfTSLP amplification and the picture show the corresponding amplification curve.

(D) Amplification of lfTSLP isoform from patients' samples using primer pair described in (Fornasa et al., 2015). The chart presents the raw data of Ct values for lfTSLP amplification and the picture show the corresponding amplification curve.

(E) Amplification of sfTSLP isoform from patients' samples using our own primer pair. The chart presents the raw data of Ct values for lfTSLP amplification and the picture show the corresponding amplification curve.

The data show in panel C, D and E correspond to amplification data obtained from samples retro-transcribed at the same time, in the same conditions and ran on the same qPCR plaque.
