# Supplementary material for: The Expression of the Short Isoform of Thymic Stromal Lymphopoietin in the Colon Is Regulated by the Nuclear Receptor Peroxisome Proliferator Activated Receptor-Gamma and Is Impaired during Ulcerative Colitis
Source: Front Immunol. 2017 Sep 4;8:1052. doi: 10.3389/fimmu.2017.01052 (PMC5591373; doi:10.3389/fimmu.2017.01052)
Supplement: Supplementary file 2 [file Data_Sheet_2.PDF]

SUPPLEMENTARY FIGURE S2

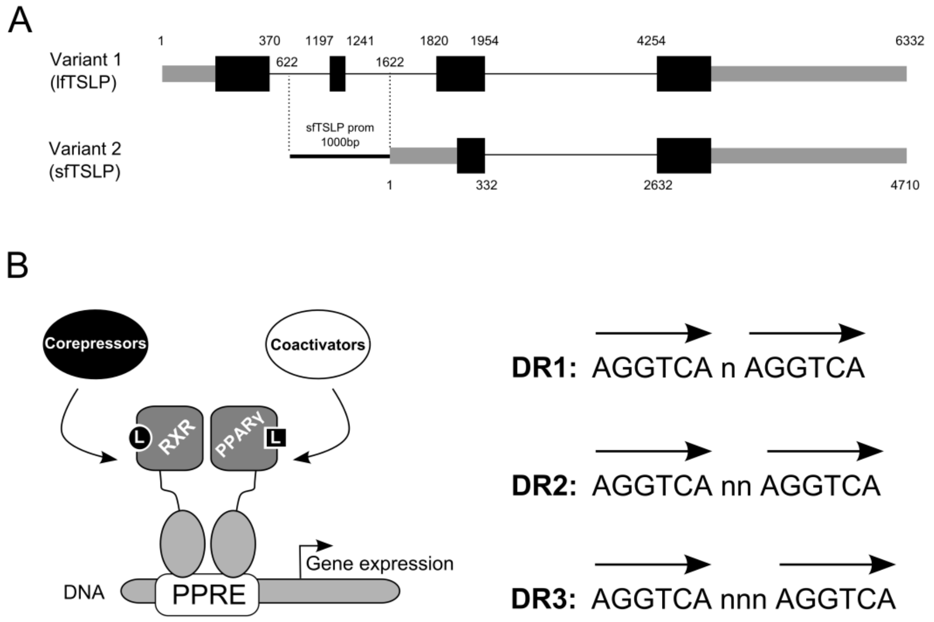

C

TGTCTATCCTTTGCTAAAGTGTTACTCTGACTATGCTGTAAAAAGCAGGAGGTAAGAGCTTAAGAAAGAGGAGTAAAAGA
GATAATTCTCATGAGATAAACTCTAAGGATTGATGCTGTGCTCCAGGTCTCTCCAGTGTTTTAGATGTTTCAGGATGCTA
TTTATTACAGAATATGGTGTACTTGGAATTTTTTTTAACATACAGTAGTAATCAT**TTTCCTGATTAACCT**AATTTCTAGA
CAGAGTTTGCATTCATGAATGGCCACAGTACAGATGCGGACATCCAAAGGATGGCATTATTACTCACAAGCATAGTGCTA
TGTGCAGTTATGGCTTGAGGGAAGGGAGGGGGGAGGTCGCCCTCTGAGACCTGAACCTTTTGGTGTGGTTTCAAGCACTA
ACCAGCACTATCTAATGGCTATTTCACTGCCTTGTCAATGACATAGGAAAAAGGTACCTGAGTGGAAACTGTTTTCAGGG
CACCTTTAAAGCCTGGGAGCAAAGGGTGGAGGGATGATTTTCCTTGTGGACTTAAAAGTCTT**TACCCTCTTTGTCCT**ATT
TTTCTTTCTTCCAGACCAA**AAGTACCGAGTTCA**ACAACACCGTCTCTTGTAGCAATCGGGTGAGTAGAGAGTTCAGTGCT
GCTGGCTTTCTCCAGGGAGACGCCAGGCATTTTGGAGAGGGAGTATCCTGCTACGTGCAGAACTCCGAGAGGTGCCTGGG
CTCCGGGACGCCGCCGCC**GGGGGAAAGGGGA**CATCTGGGCTGTCAGAGCGGGGCTGCGCCTAGCTTGGGACAACACTTCT
GTTCCAATTTAGGGAGAGGAAGTCTCTATCCGGAGGAAAGGCAAATTGGGAACTGGGACGAGGGAACGTTGTTAGGGGCA
CCACCTGCTGGGGTCCGGCGCCTCCGCGCTCGGGCTCGGAATTTTGGCAGCCTCCGCCCCCTGGAGACTTGGGAGGAGCG
AGCGTGGGTGACAGTCTTTTCGCGACGAGTGCCCTCCGCC**acc**ctcgccacgcccctgctcccccgcggttggttcttcct
tgctctactcaaccctgacctcttctctctgactctcgacttgtgttccccgctcctccctgaccttcctcccctcccctt
tcactcaattctcaccaactctttctctctctggtgttttctccttttctcgtaaactttgccgcctatgagcagccacat
tgccttactgaaatccagagcctaaccttcaatcccaccgccggctgcgcgtcgctcgccaaagaaATGttcgccATGaaa
actaaggctgccttag…

Supplementary figure S2: *In silico* identification of PPRE in the sfTSLP gene promoter.

(A) Schematic representation of the structural organization of the *tslp* gene with its two isoforms. Variant 1 represents the long form TSLP (lfTSLP) and variant 2 represents the short isoform (sfTSLP). As in Figure 3A, the rectangles represent the exons, the gray parts corresponding to the untranslated regions and the black parts corresponding to the translated regions. Numbers represent nucleotide count for each exon with the nucleotide at the transcription start site for each isoform is referred as nucleotide +1. The 1000 bp promoter region of sfTSLP corresponds to a region of the lfTSLP gene including nucleotides +622 to +1622 which encompass exon 2.

(B) PPARγ heterodimerizes with the 9-cis-retinoic acid receptor (RXR) to bind to response elements (PPREs) located in target genes to activate transcription. PPARγ-RXR is a permissive heterodimer, as the transcriptional activity of the dimeric complex can be activated by both PPARγ and RXR agonists. PPARγ-RXR heterodimer recognizes PPRE composed of a direct repetition of the consensus sequence AGGTCA separated by one (DR1), two (DR2) or three (DR3) nucleotide(s). Figure adapted from (Bertin et al., 2013).

(C) We used bioinformatics tools to find PPRE in the human sfTSLP promoter gene (1,000 bp upstream to the transcription start point ; represented by uppercase letters). Two different programs were used: NUBIScan (Podvinec et al., 2002) and PPRE Search (http://www.classicrus.com/PPRE/) (Venkatachalam et al., 2009). The putative DR1 identified in the 1,000 bp sequence of the sfTSLP gene promoter are denoted in green and

underlined bold letters. The putative DR2 identified are denoted in blue and underlined bold letters. The putative DR3 identified are denoted in red and underlined bold letters. The nucleotides in lowercase denote the transcribed region with the transcription start point indicated by black, bold and underlined nucleotides. At the end of the sequence, translated nucleotides are underlined and the two ATG initiation codons are in red.
