# Supplementary material for: The Expression of the Short Isoform of Thymic Stromal Lymphopoietin in the Colon Is Regulated by the Nuclear Receptor Peroxisome Proliferator Activated Receptor-Gamma and Is Impaired during Ulcerative Colitis
Source: Front Immunol. 2017 Sep 4;8:1052. doi: 10.3389/fimmu.2017.01052 (PMC5591373; doi:10.3389/fimmu.2017.01052)
Supplement: Supplementary file 3 [file Data_Sheet_3.PDF]

SUPPLEMENTARY FIGURE S3

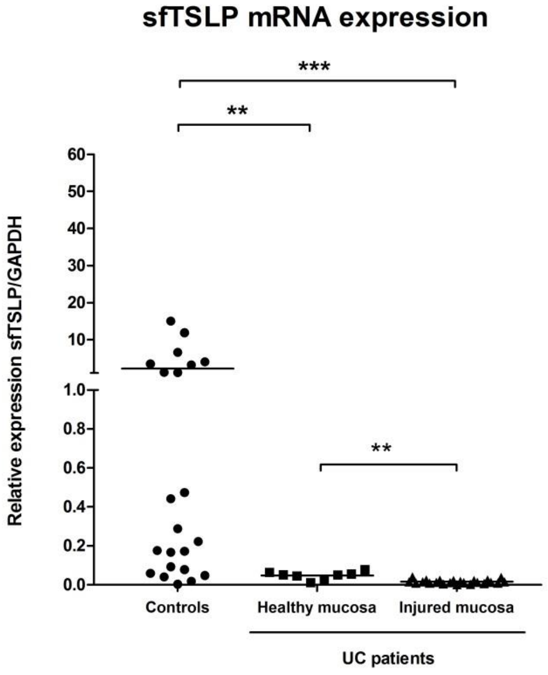

Supplementary figure S3: sfTSLP expression in colonic samples of control specimens and UC patients using primer pair described in (Fornasa et al., 2015).

Quantitative expression of sfTSLP mRNA was assessed by qPCR and normalized to GAPDH level. Horizontal bar indicates the mean value. **P<0.01, ***P<0.001.
