# Supplementary figures and images for: The Expression of the Short Isoform of Thymic Stromal Lymphopoietin in the Colon Is Regulated by the Nuclear Receptor Peroxisome Proliferator Activated Receptor-Gamma and Is Impaired during Ulcerative Colitis
Source: Front Immunol. 2017 Sep 4;8:1052. doi: 10.3389/fimmu.2017.01052 (PMC5591373; doi:10.3389/fimmu.2017.01052)

## SUPPLEMENTARY FIGURE S4

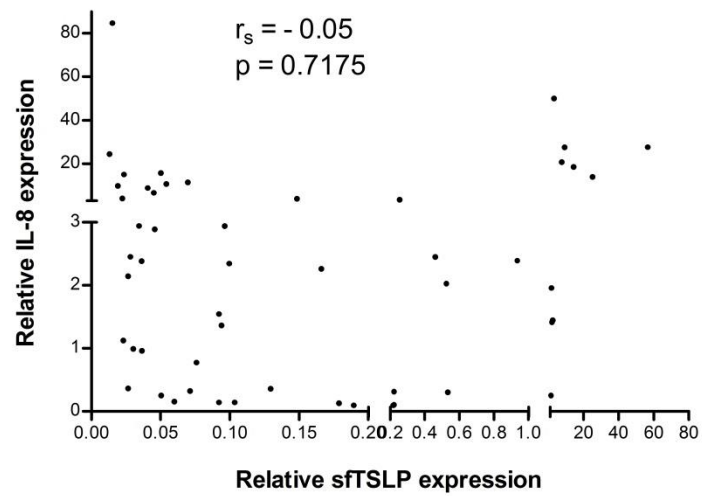

Supplement: Supplementary file 4 [file Data_Sheet_4.PDF]
