# Supplementary material for: The Expression of the Short Isoform of Thymic Stromal Lymphopoietin in the Colon Is Regulated by the Nuclear Receptor Peroxisome Proliferator Activated Receptor-Gamma and Is Impaired during Ulcerative Colitis
Source: Front Immunol. 2017 Sep 4;8:1052. doi: 10.3389/fimmu.2017.01052 (PMC5591373; doi:10.3389/fimmu.2017.01052)
Supplement: Supplementary file 5 [file Table_1.PDF]

SUPPLEMENTARY TABLE S1

Oligonucleotides used in the study

Genes	Forward sequences	Reverse sequences
GAPDH	5’-GACACCCACTCCTCCACCTTT-3’	5’-TTGCTGTAGCCAAATTCGTTGT-3’
PPARγ	5’-GCTGTCATTATTCTCAGTGGAGAC-3’	5’-GTCTTCTTGATCACATGCAGTAG-3’
TSLP	5’-TGCCTTAGCTACTGGTGCC-3’	5’-ACGCCACAATCCTTGTAATTG-3’
TSLP 1	5’-GGGCTGGTGTTAACTTACGACTTCA-3’	5’-ACTCGGTACTTTTGGTCCCACTCA-3’
TSLP 2	5’-CGTAAACTTTGCCGCCTATGA-3’	5’-TTCTTCATTGCCTGAGTAGCATTTAT-3’
LfTSLP (Fornasa et al)[4].	5’-CACCGTCTCTTGTAGCAATCG-3’	5’-TAGCCTGGGCACCAGATAGC-3’
SfTSLP (Fornasa et al.)	5’-CCGCCTATGAGCAGCCAC-3’	5’-CCTGAGTAGCATTTATCTGAG-3’
IL-8	5’-AAGGAACCATCTCACTGTGTGTAAAC-3’	5’-AAATCAGGAAGGCTGCCAAGA-3’
IL-1β	5’-TTGTTGAGCCAGGCCTCTCT- 3’	5’-CCAAATGTGGCCGTGGTT-3’
LT β	5’-TATCACTGTCCTGGCTGTGC-3’	5’-GCTTCTGAAACCCCAGTCCT-3’
TNF α	5’-ATCAATCGGCCCGACTATCTC-3’	5’-ACAGGGCAATGATCCCAAAGT-3’
CCL 28	5’-AATGCAGCAGAGAGGACTCG-3’	5’-ATGTGAAACCTCCGTGCAAC-3’
BAFF	5’-CGTTCAGGGTCCAGAAGAAA-3’	5’-AAGCTGAGAAGCCATGGAAC-3’
IL-10 Rα	5’-AACTGGACCGTCACCACCAC-3’	5’-GGCCTGGGTAGCTGAATCTT-3’
CCL 20	5’-GTGCTGCTACTCCACCTCTG-3’	5’-CGTGTGAAGCCCACAATAAA-3’
TGF β	5’-CAAGCAGAGTACACACAGCAT-3’	5’-TGCTCCACTTTTAACTTGAGCC-3’
RALDH 1	5’-GCACGCCAGACTTACCTGTC-3’	5’-CCACTCACTGAATCATGCCA-3’
IL-13 Rα 1	5’-TCCCTCCAATTCCTGATCCT-3’	5’-AGTCGGTTTCCTCCTTGGTT-3’
IL-22 R	5’-AAATCATGATGGTGCCAAGG-3’	5’-TTAGAGCTCCAGGTCAACCG-3’
IL-10 Rβ	5’-GGGAACCTGACTTTCACAGC-3’	5’-CCTGACTCTCAAGGTGTGGTC-3’
3a – 4b	5’-CAGAATATGGTGTACTTGGAA-3’	5’-GTACTGTGGCCATTCATGAATGC-3’
