# Supplementary material for: The Expression of the Short Isoform of Thymic Stromal Lymphopoietin in the Colon Is Regulated by the Nuclear Receptor Peroxisome Proliferator Activated Receptor-Gamma and Is Impaired during Ulcerative Colitis
Source: Front Immunol. 2017 Sep 4;8:1052. doi: 10.3389/fimmu.2017.01052 (PMC5591373; doi:10.3389/fimmu.2017.01052)
Supplement: Supplementary file 6 [file Table_2.PDF]

SUPPLEMENTARY TABLE S2

Control subjects (n=22)

Sex distribution	
Number of males (% of males)	12/22 (54.5%)
Age at inclusion	57.5 ± 12.9
Pathology	
Polyposis	4/22 (18.2%)
Adenocarcinoma	4/22 (18.2%)
Diverticulitis	13/22 (59.1%)
Toxic megacolon	1/22 (4.5%)

Ulcerative colitis (n=23)

Sex distribution	
Number of males (% of males)	09/23 (39.1%)
Age at inclusion	42.6 ± 13.6
Age at diagnosis	35,2 ± 12.2 *(missing data n=2)*
Disease extent	
Left sided colitis	4/23 (17.4%)
Extended colitis	19/23 (82.6%)
Treatments at inclusion	
Aminosalicylates	6/23 (26.1%)
Corticoids	6/23 (26.1%)
Immunosuppressants	2/23 (8.7%)
Anti-TNF	8/23 (34.8%)
None	1/23 (4.3%)
Missing data	1/23 (4.3%)
Type of surgery	
Colectomy	17/23 (74%)
Protectomy	3/23 (13%)
Sigmoidectomy	3/23 (13%)
